# Treatment Adherence in Inflammatory Bowel Disease Patients from Argentina: A Multicenter Study

**DOI:** 10.1155/2020/4060648

**Published:** 2020-01-17

**Authors:** Juan Lasa, Gustavo Correa, Claudia Fuxman, Laura Garbi, Maria Eugenia Linares, Pablo Lubrano, Astrid Rausch, Martin Toro, Martin Yantorno, Ignacio Zubiaurre, Laurent Peyrin-Biroulet, Pablo Olivera

**Affiliations:** ^1^Gastroenterology Department, Hospital Britanico de Buenos Aires, Buenos Aires, Argentina; ^2^Gastroenterology Section, Department of Internal Medicine, Centro de Educación Médica e Investigaciones Clínicas (CEMIC), Buenos Aires, Argentina; ^3^Gastroenterology Department, Hospital Interzonal General de Agudos “General José de San Martín”, La Plata, Argentina; ^4^Gastroenterology Department, Hospital Universitario Fundacion Favaloro, Buenos Aires, Argentina; ^5^Gastroenterology Department, Hospital de Clínicas “José de San Martín”, Buenos Aires, Argentina; ^6^Gastroenterology Department, Sanatorio Mater Dei, Buenos Aires, Argentina; ^7^Gastroenterology Department, Hospital Universitario, Universidad Nacional de Cuyo, Mendoza, Argentina; ^8^INSERM U954 and Department of Hepatogastroenterology, Nancy University Hospital, Université de Lorraine, Vandoeuvre-lès-Nancy, France

## Abstract

**Methods:**

A multicenter cross-sectional study involving seven referral centers from three cities of Argentina was undertaken. Patients with a diagnosis of ulcerative colitis (UC), Crohn's disease (CD), or indeterminate colitis (IBDU/IC) were invited to answer an anonymous survey, which included a 5-point Likert scale to evaluate adherence to therapies. Independent variables associated with inadequate adherence were evaluated.

**Results:**

Overall, 447 UC/IBDU and 135 CD patients were enrolled. Median age was 37 years (range 21-72); 39.8% were male; median time from diagnosis was 6 years (0.5-35). 91.4% were under treatment with at least one oral medication; 50.3% of patients reported inadequate adherence to oral medications. Patients with UC/IBDU had a lower risk of inadequate adherence when compared to patients with CD (OR 0.57 (0.37-0.87)). 21.8% reported inadequate adherence to biologics; subcutaneous administration was significantly associated with inadequate adherence to biologics (OR 4.8 (1.57-14.66)).

**Conclusion:**

Inadequate treatment adherence is common among patients with IBD, and potentially modifiable factors were identified.

## 1. Introduction

Inflammatory bowel disease (IBD) comprises mainly two chronic, immune-mediated, and potentially disabling conditions: ulcerative colitis (UC) and Crohn's disease (CD) [1, 2].

Currently, medical treatment consists in the administration of medications that induce a modulation of the immune system at the gastrointestinal level, halting inflammatory activity in IBD patients [3]. Given that these are chronic diseases characterized by periods of flare and remission, maintenance therapy is usually needed lifelong [4].

Many medications, especially biologics, need parenteral administration, which can be bothersome in patients requiring therapy for long periods of time [5]. Several factors may hamper adherence to prescribed treatments in IBD patients, and a noncompliant behavior has been associated with a higher risk of disease relapse, reduced quality of life, increased healthcare costs, and a higher risk of disability and complications [6–10].

Previous studies have described the prevalence of inadequate adherence in IBD patients, as well as factors associated with this behavior [11–18]. Notwithstanding this, these results may be influenced by many factors, including the method used to define inadequate adherence, data collection, and the studied population, especially with regard to idiosyncrasy and characteristics of different health systems. Disease-related knowledge of IBD patients may differ across cultures, and it has been related to quality of life, coping skills, and treatment adherence [19–21]. Adherence measurement and identification of factors associated with inadequate adherence in developing countries are imperative, in order to allow interventions that ultimately improve outcomes in IBD patients, and reduce healthcare costs. To the best of our knowledge, no previous study has addressed treatment adherence of IBD patients in Argentina.

The aim of the present study was to determine inadequate adherence to oral and parenteral treatments among IBD patients from Argentina and to identify factors associated with nonadherence.

## 2. Methods

### 2.1. Study Design and Population

A multicenter cross-sectional study involving seven referral centers from three cities of Argentina was undertaken. A survey to evaluate treatment adherence was designed, which included several questions using a 5-point Likert scale design for the evaluation of adherence to the following medications: 5-aminosalicylates, thiopurines, and biologics.

The questionnaire also included the following variables: age, gender, years from diagnosis, education level, type of medical insurance, smoking, history of surgery and/or hospitalization due to IBD, number of annual visits to a gastroenterologist, patient's perception of easy communication with the gastroenterologist, use of e-mail/text messages for consultation, overall satisfaction with medical attention, and use of other chronic medications.

The survey was placed in an online platform (Google Forms, http://www.google.com/forms, Mountain View, CA, USA), which was only accessible by invitation. Invitations were sent via e-mail, text messages, and social media to patients between February 1 and April 10, 2018. Patients older than 18 years old, with an established diagnosis of UC, CD, or IBDU/IC, under pharmacologic treatment were invited to take the survey. Given the expected small number of patients with IBDU/IC, and the similar treatments and clinical characteristics with UC patients, these two groups were analyzed together. Exclusion criteria were ongoing hospitalization due to severe IBD flare, history of dementia or neurodegenerative disorders, and lack of Internet access.

The primary outcome of interest was the proportion of patients with inadequate adherence to oral medications (i.e., 5-aminosalicylates and thiopurines) and biologics. Adherence to medications was assessed using a 5-point Likert scale asking how often did patients forget to take their medications (*never–rarely–sometimes–often–always*). A secondary outcome was to identify factors independently associated with low adherence.

The proportion of patients with inadequate adherence to treatment—the primary endpoint—was defined as the number of patients who answered the options “rarely” or “sometimes” or “often” or “always” to the question “how often do you miss medication intake?” This definition was used to define inadequate adherence to 5-aminosalicylates and thiopurines and biologics separately.

### 2.2. Statistical Analysis

Statistical analysis was carried out using the Stata program (v11.1, StataCorp, College Station, Texas, USA). A global inadequate adherence to treatment prevalence of 25% was assumed; considering an alpha error of less than 5% and a power of 80%, 288 complete surveys would be required. Assuming that the proportion of subjects invited to participate who satisfactorily complete the survey is 75%, it would be required to send the invitation to participate to at least 384 patients. Categorical variables were described as percentages, whereas numerical variables were described as median with range. Univariate analyses to identify variables associated with lack of adherence to IBD medications were performed; we estimated the odds ratios (OR) with their corresponding 95% confidence intervals (CI95%). Variables with a *p* value of less than 0.1 were included in multivariate analyses using a logistic regression model to determine their association with inadequate treatment adherence.

## 3. Results

### 3.1. Baseline Characteristics

**Figure 1:**
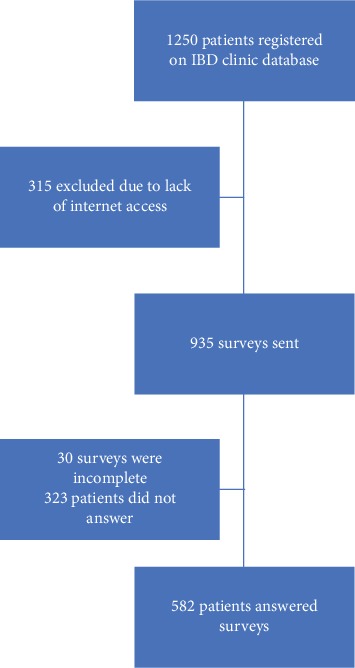
Flow chart showing patient selection process.

Figure 1 shows the patient selection process. Overall, 582 patients with IBD were finally enrolled; 429 patients with UC (73.7%), 135 patients with CD (23.2%), and 18 patients with IBDU/IC (3.1%). Median age was 37 years (range 21-72); 39.8% were male; median time from diagnosis was 6 years (range 0.5-35).  Table 1 shows the main demographic and clinical characteristics of the patients with IBD that completed the survey.

### 3.2. Medications

Table 2 summarizes the medications used by the surveyed patients, according to their diagnosis. Most of the patients received at least one oral medication (overall 532/582, 91.4%; CD 84.4%; UC/IBDU 93.5%). The use of oral 5-aminosalicylates was high in both CU/IBDU and CD (80.3% and 85.9%, respectively, *p* = 0.1). There was a nonsignificant trend towards an increased use of thiopurines (56.3% vs. 35.8%, *p* = 0.08) and biologics (32.6% vs. 25.1%, *p* = 0.06) in the CD group compared to the UC/IBDU group.

### 3.3. Adherence

Overall, adherence to oral medications was regarded as inadequate according to the above-mentioned definition in 50.3% of patients, with a statistically significant difference between the UC/IBDU group and the CD group (47.4% vs. 61.4%, *p* = 0.01).

When considering adherence to oral 5-aminosalicylates, inadequate adherence was seen in 52.2% of patients (51.2% in UC/IBDU vs. 53.8% in CD, *p* = 0.8; OR 0.8 (0.5–1.2)). After a univariate and multivariate analysis (Table 3), variables associated with inadequate adherence to oral 5-aminosalicylates were use of chronic medication for other indication than IBD (OR 1.5 (1–2.2)), whereas perception of easy contact with the gastroenterologist was inversely associated with such event (OR 0.4 (0.2–0.8)).

Inadequate thiopurine adherence was seen in 40.2% of patients (37.4% in UC/IBDU vs. 59.3% in CD, *p* = 0.02). Inadequate adherence to oral 5-aminosalicylates was significantly higher than to thiopurines (OR 2.3 (1.7–3.3)). After a univariate and multivariate analysis (Table 4), variables associated with inadequate adherence to thiopurines were smoking (OR 3.7 (1.5–9.1)), whereas perception of easy contact with the gastroenterologist was inversely associated with such event (OR 0.6 (0.2–0.9)).

When considering adherence to biologics, inadequate adherence was seen in 15.3% of patients (19.5% in UC/IBDU vs. 24% in CD, *p* = 0.6). The risk of inadequate adherence to biologics was significantly lower than to oral 5-aminosalicylates (OR 4.1 (2.7–6.3)) and thiopurines (OR 1.7 (1.1–2.7)). After a univariate analysis (Table 5), we did not find variables associated with inadequate adherence to biologics in general. However, when evaluating adherence to subcutaneous versus intravenous biologics, the proportion of inadequate adherence to subcutaneous biologics (i.e., adalimumab, golimumab, and certolizumab pegol) was significantly higher than to intravenous biologics (i.e., infliximab and vedolizumab) (28.5% versus 7.7%; OR 4.8 (1.5–14.6)).

The most frequent self-reported reason of inadequate adherence was forgetfulness of administration for both oral and parenteral medications (Table 6). Fear of side effects was the least frequent reason of inadequate adherence to biologics (8%).

## 4. Discussion

To the best of our knowledge, this is the first study evaluating treatment adherence in IBD population in a Latin-American country. The primary aim of the present study was to determine inadequate adherence to 5-aminosalicylates, thiopurines, and biologics in patients with IBD from Argentina from different backgrounds.

Overall, more than a half of the surveyed patients have inadequate adherence to oral 5-aminosalicylates, while two-fifths and one-fifth of patients have inadequate adherence to thiopurines and biologics, respectively. Inadequate adherence to oral 5-aminosalicylates found in our study is in line with previous reports, which ranges widely between 38% and 60%, depending on the studied population and the method used to define adherence [11, 13, 22–25]. We found a significantly lower odds of inadequate adherence to thiopurines compared to oral 5-aminosalicylates, a trend previously seen in the literature [26–28]. However, the inadequate adherence to thiopurines seen in our study was numerically higher than previous reports, which ranged between 32% and 9% [14, 26, 27, 29, 30].

The prevalence of inadequate adherence to biologic therapy was again in line with previous reports and was mainly driven by infliximab and adalimumab use, since the number of patients under golimumab, certolizumab pegol, and vedolizumab was relatively small. In a systematic review, Lopez et al. described a pooled adherence prevalence to anti-TNF therapy of 82.6% [18]. A recent retrospective cohort study by Wentworth et al. reported a 24-month adherence prevalence to biologic therapy of 66% [17]. Interestingly, they described for the first time an adherence prevalence to vedolizumab of 82%, which was numerically higher than to anti-TNF agents (which ranged between 50% and 70%) [17]. Unfortunately, there is no consensus on how to define adherence to biologics, and there is no validated measurement tool in IBD population. Previous studies used tools validated for other conditions, such as the medication possession ratio or the medication refill adherence [31].

Factors associated with inadequate adherence to oral 5-aminosalicylates and thiopurines were identified. After univariate and multivariate analyses, perception of ease in contacting the gastroenterologist in case of need was a protective factor for inadequate adherence to both oral 5-aminosalicylates and thiopurines. Quality of care and high patient satisfaction have previously been linked with high treatment adherence rates in IBD patients [32]. This finding underscores the importance of IBD nurses in the IBD multidisciplinary team, in which one of their key role is accessible care [33]. Due to several reasons, in Argentina and other developing countries, the role of IBD nurse has not been extensively implemented, which is frequently translated in a high workload to the physicians. Concomitant use of medications for other indication than IBD was a risk factor for inadequate adherence to oral 5-aminosalicylates. The most frequently self-reported reason for inadequate adherence to oral medications was forgetfulness, so taking several medications could preclude from adhering to them. We found that smoking was a risk factor of inadequate adherence to thiopurines, a phenomenon also seen in other conditions [34]. Unfortunately, we were not able to identify factors associated with adherence to biologics.

The main strength of this study is that it included a significant number of participants and was well powered to define differences in adherence rates between treatments. We included patients from different regions of Argentina, from different backgrounds and social levels, followed in both public and private institutions. The present study represents the first study evaluating adherence in IBD population in our country, in which the health system, access to care, and idiosyncrasy of patients are particularly different from other regions of the world [35].

Our study has several limitations. Firstly, as in other studies that evaluated adherence based on patients' surveys, the results might be prone to recall bias. What is more, although the survey was anonymous, patients might have given answers based on their perceived expectations of their gastroenterologist. This may explain the very high rate of satisfaction with their gastroenterologist's care. Secondly, given that only patients with regular access to the Internet were invited to take the survey, selection bias towards younger population may have occurred. Thirdly, a Likert scale was used to evaluate adherence to oral and biologic therapies, using an arbitrary cut-off for inadequate adherence. Although validated tools for evaluation of adherence to oral medications exist, there is lack of tools and consensus definition of inadequate adherence to biologic therapies. Until such a tool and definition becomes available, most of the studies will face this limitation. Fourthly, it was not possible to define specific disease characteristics, such as inflammatory activity, Montreal classification, and line of therapy.

Inadequate adherence has a negative impact on disease outcomes and healthcare costs in IBD [36]. Knowing adherence rates in a given population and describing patients at risk of inadequate adherence are of outmost importance. We found that inadequate adherence in Argentinean IBD patients is similar to those of patients from other regions of the world, in spite of differences in health systems and access to care. Potentially modifiable factors associated with an increased odd of inadequate adherence to medications were identified, and efforts should be directed towards improving medication adherence in IBD patients.

## Figures and Tables

**Table 1:** Characteristics of participants.

Variable	Median (IQR), % (*n*)
Age	37 (21-72)
Gender (% male)	39.86
Diagnosis	
Ulcerative colitis	73.71 (429/582)
Crohn's disease	23.20 (135/582)
IBDU	3.09 (18/582)
Years since diagnosis	6 (1-35)
Residence in Argentina	86.25 (502/582)
Level of education	
Primary	7.56 (44/582)
Secondary	33.51 (195/582)
Tertiary	58.93 (343/582)
Lives alone	17.53 (102/582)
Disability certificate	24.57 (143/582)
Health coverage	
Union-funded health insurance	46.56 (271/582)
Private health insurance	45.88 (267/582)
No health coverage	7.56 (44/582)
Percentage of reimbursement of medications' costs	
100%	31.96 (186/582)
70%	36.60 (213/582)
40%	12.89 (75/582)
Other	18.56 (108/582)
Smoking	12.71 (74/582)
History of IBD-related surgery	13.40 (78/582)
History of hospitalization due to IBD	51.55 (300/582)
Number of consultations to the gastroenterologist in the last year	
>5/year	44.50 (259/582)
≤4/year	55.50 (323/582)
Satisfaction with the number of consultations with the gastroenterologist	83.33 (485/582)
Perception of easy contact with gastroenterologist in case of need	84.02 (489/582)
Satisfaction with gastroenterologist's care	92.78 (540/582)
Communication with gastroenterologist via email or text messages	70.79 (412/582)
Use chronic medication for other indication than IBD	31.27 (182/582)
Taking other medications affect adherence to IBD treatment	17.42 (23/182)

IBD: inflammatory bowel disease; IBDU: inflammatory bowel disease unclassified.

**Table 2:** Comparative use of chronic medications between enrolled UC/IBDU and CD patients.

	UC/IBDU (*N* = 447), %	CD (*n* = 135), %	OR (CI95%)	*p*
Oral 5-ASA	80.31 (359/447)	85.93 (116/135)	0.67 (0.39-1.14)	0.1
Rectal 5-ASA	22.37 (100/447)	16.3 (22/135)	1.48 (0.89-2.46)	0.1
Oral budesonide	3.36 (15/447)	2.22 (3/135)	1.52 (0.43-5.36)	0.5
Thiopurines	35.8 (160/447)	56.29 (76/135)	0.69 (0.45-1.05)	0.08
Methotrexate	0.89 (4/447)	0.74 (1/135)	1.21 (0.13-10.93)	0.8
Prednisone	8.72 (39/447)	11.85 (16/135)	0.71 (0.38-1.32)	0.2
Biologics (all)	24.83 (111/447)	33.33 (45/135)	0.69 (0.45-1)	0.06
Infliximab	9.17 (41/447)	11.11 (15/135)	0.8 (0.43-1.51)	0.5
Adalimumab	13.65 (61/447)	19.26 (26/135)	0.66 (0.39-1.1)	0.1
Vedolizumab	0.89 (4/447)	1.48 (2/135)	0.6 (0.1-3.32)	0.5
Certolizumab	0	1.37 (2/135)	N/A	0.4
Golimumab	1.12 (5/447)	0	N/A	0.1

UC: ulcerative colitis; CD: Crohn's disease; IBDU: inflammatory bowel disease unclassified; OR: odds ratio; CI: confidence interval; 5-ASA: 5-aminosalicylates.

**Table 3:** Univariate and multivariate analysis of variables associated with inadequate adherence to 5-aminosalicylates.

	Inadequate adherence group (*N* = 248)	Adequate adherence group (*n* = 227)	Univariate analysis		Multivariate analysis	
			OR (CI95%)	*p*	OR (CI95%)	*p*
Gender (% male)	39.91 (99/248)	40.09 (91/227)	0.9 (0.68-1.43)	0.9		
Age, median (IQR)	37 (21-72)	37 (22-68)	N/A	0.9		
Years since diagnosis, median (IQR)	6 (1-35)	6.5 (1-34)	N/A	0.7		
Primary level of education (%)	8.06 (20/248)	7.49 (17/227)	1.08 (0.5-2.12)	0.8		
Lives alone (%)	20.56 (51/248)	15.42 (35/227)	1.42 (0.8-2.28)	0.14		
Disability certificate (%)	24.19 (60/248)	22.47 (51/227)	1.1 (0.72-1.68)	0.6		
Health coverage (% union-funded health insurance)	43.15 (107/248)	47.58 (108/227)	0.83 (0.58-1.21)	0.3		
Smoking (%)	13.31 (33/248)	12.33 (28/227)	1.09 (0.63-1.87)	0.7		
History of IBD-related surgery (%)	11.69 (29/248)	16.74 (38/227)	0.65 (0.39-1.12)	0.1	0.77 (0.45–1.31)	0.3
History of hospitalization due to IBD (%)	47.98 (119/248)	52.42 (119/227)	0.83 (0.58-1.2)	0.3		
>5 consultations in the last year (%)	43.95 (109/248)	45.37 (103/227)	0.94 (0.65-1.35)	0.7		
Satisfaction with number of consultations (%)	78.63 (195/248)	88.11 (200/227)	0.49 (0.29-0.82)	0.006	0.61 (0.35-1.06)	0.08
Perception of easy contact (%)	**79.84 (194/248)**	**91.63 (208/227)**	**0.36 (0.2-0.64)**	**0.001**	**0.43 (0.23–0.81)**	**0.01**
Satisfaction with gastroenterologist's care (%)	92.34 (229/248)	94.71 (215/227)	0.67 (0.32-1.42)	0.3		
Communication with gastroenterologist via email or text messages (%)	66.53 (165/248)	77.09 (175/227)	0.59 (0.39-0.89)	0.01	0.79 (0.51 – 1.24)	0.3
Use chronic medication for other indication than IBD (%)	**37.9 (94/248)**	**27.75 (63/227)**	**1.59 (1.07-2.34)**	**0.01**	**1.49 (1.01–2.25)**	**0.03**

IBD: inflammatory bowel disease; IQR: interquartile range; OR: odds ratio; CI: confidence interval. Statistically significant associated variables after the multivariate analysis are shown in bold.

**Table 4:** Univariate and multivariate analysis of variables associated with inadequate adherence to thiopurines.

	Inadequate adherence group (*N* = 95)	Adequate adherence group (*n* = 141)	Univariate analysis		Multivariate analysis	
			OR (CI95%)	*p*	OR (CI95%)	*p*
Gender (% male)	46.32 (44/95)	39.72 (56/141)	1.31 (0.77-2.22)	0.3		
Age, median (IQR)	37 (22/71)	36 (21/70)	N/A	0.8		
Years since diagnosis, median (IQR)	6 (1-37)	6 (1-31)	N/A	0.8		
Primary level of education (%)	9.47 (9/95)	6.38 (9/141)	1.53 (0.58-4.03)	0.4		
Lives alone (%)	20 (19/95)	17.02 (24/141)	1.22 (0.62-2.38)	0.5		
Disability certificate (%)	31.58 (30/95)	22 (31/141)	1.63 (0.9-2.96)	0.09		
Health coverage (% union-funded health insurance)	49.47 (47/95)	50.35 (71/141)	0.96 (0.57-1.62)	0.9		
Smoking (%)	**18.95 (18/95)**	**5.67 (8/141)**	**3.88 (1.57-9.56)**	**0.01**	**3.75 (1.53–9.17)**	**0.004**
History of IBD-related surgery (%)	8.42 (8/95)	10.64 (15/141)	0.77 (0.31-1.9)	0.5		
History of hospitalization due to IBD (%)	46.32 (44/95)	55.32 (78/141)	0.69 (0.41-1.17)	0.17		
>5 consultations in the last year (%)	46.32 (44/95)	43.26 (61/141)	1.13 (0.7-1.91)	0.6		
Satisfaction with number of consultations (%)	77.89 (74/95)	86.52 (122/141)	0.54 (0.27-1.09)	0.08	0.64 (0.3–1.34)	0.24
Perception of easy contact (%)	**82.11 (78/95)**	**90.07 (127/141)**	**0.50 (0.23-1.09)**	**0.07**	**0.66 (0.27–0.9)**	**0.05**
Satisfaction with gastroenterologist's care (%)	90.53 (86/95)	95.74 (135/141)	0.42 (0.14-1.24)	0.1		
Communication with gastroenterologist via email or text messages (%)	66.32 (63/95)	77.3 (109/141)	0.58 (0.32-1.03)	0.06	0.71 (0.36–1.36)	0.3
Use chronic medication for other indication than IBD (%)	29.47 (28/95)	29.08 (41/141)	1.02 (0.57-1.81)	0.9		

IBD: inflammatory bowel disease; IQR: interquartile range; OR: odds ratio; CI: confidence interval. Statistically significant associated variables after the multivariate analysis are shown in bold.

**Table 5:** Univariate analysis of variables associated with inadequate adherence to biologics.

	Inadequate adherence group (*N* = 34)	Adequate adherence group (*n* = 122)	Univariate analysis	
			OR (CI95%)	*p*
Gender (% male)	35.29 (12/34)	42.62 (52/122)	0.74 (0.35-1.58)	0.4
Age, median (IQR)	37 (22-69)	37 (21-72)	N/A	0.5
Years since diagnosis, median (IQR)	6 (1-33)	6 (1-26)	N/A	0.9
Primary level of education (%)	5.88 (2/34)	9.01 (11/122)	0.56 (0.12-2.62)	0.4
Live alone (%)	11.76 (4/34)	20.49 (25/122)	0.48 (0.15-1.46)	0.2
Disability certificate (%)	29.41 (10/34)	30.32 (37/122)	0.87 (0.39-1.94)	0.7
Health coverage (% union-funded health insurance)	44.11 (15/34)	51.64 (63/122)	0.74 (0.35-1.54)	0.4
Smoking (%)	11.76 (4/34)	13.93 (17/122)	0.96 (0.33-2.73)	0.9
History of IBD-related surgery (%)	23.52 (8/34)	18.03 (22/122)	1.39 (0.56-3.49)	0.6
History of hospitalization due to IBD (%)	55.88 (19/34)	58.19 (71/122)	0.87 (0.42-1.83)	0.7
>5 consultations in the last year (%)	70.59 (24/34)	63.93 (78/122)	1.43 (0.64-3.2)	0.3
Satisfaction with number of consultations (%)	76.47 (26/34)	82.78 (101/122)	0.71 (0.29-1.73)	0.4
Perception of easy contact (%)	82.35 (28/34)	85.24 (104/122)	0.84 (0.31-2.24)	0.7
Satisfaction with gastroenterologist's care (%)	88.23 (30/34)	90.98 (111/122)	0.76 (0.23-2.49)	0.6
Communication with gastroenterologist via email or text messages (%)	64.71 (22/34)	70.49 (86/122)	0.83 (0.38-1.81)	0.6
Use chronic medication for other indication than IBD (%)	32.35 (11/34)	27.05 (33/122)	1.31 (0.6-2.86)	0.4

IBD: inflammatory bowel disease; IQR: interquartile range; OR: odds ratio; CI: confidence interval.

**Table 6:** Self-reported reasons of inadequate adherence.

	Oral medications	Parenteral medications
Because I forget	51.73%	34.38%
Because I feel well and I think I do not need the medication	13.56%	12.2%
Because the medication is expensive	11.73%	18.85%
Because I run out of medication before I get a new prescription	11%	17.21%
Because of fears to side effects	6.62%	8.3%
Because the medication is not available in pharmacies	5.36%	9.06%

## Data Availability

The data used to support the results included in the manuscript are gathered in a database which is accessed by the authors of the study only, in agreement with the corresponding ethics committees that have approved this study.
